# Retraction: Male breast cancer: thirteen years experience of a single center

**DOI:** 10.1186/1477-7800-6-11

**Published:** 2009-04-17

**Authors:** Sami Akbulut, Ilker Arer, Alper Kocbiyik, Mahmut Can Yağmurdur, Hamdi Karakayalı, Mehmet Haberal

**Affiliations:** 1 Department of Surgery, University Hospital of Baskent, Ankara 06490, Turkey; 2 Department of Pathology, University Hospital of Baskent, Ankara 06490, Turkey

## Retraction

This article [1] was submitted to *International Seminars of Surgical Oncology *without the knowledge or consent of either Prof Karakayali or Prof Haberal, and is being retracted for this reason. An apology is extended to Prof Karakayali and Prof Haberal as well as the editorial and publishing staff and readers of the journal.
